# Preoperative depressive symptoms predict sustained postoperative inflammation and longer hospital stay after cardiac surgery

**DOI:** 10.1016/j.bbih.2026.101306

**Published:** 2026-07-13

**Authors:** Stefan Salzmann, Frank Euteneuer, Ruben Domroese, Udo Boeken, Artur Lichtenberg, Ulrike Dinger, Clarissa Zimmermann, Malin Neumann, Ralf Schäfer, Anna Markser

**Affiliations:** a Division of Clinical Psychology and Psychotherapy, Marburg University, Marburg, Germany; b Faculty of Human Sciences, Division of Clinical Psychology and Psychotherapy, Vinzenz Pallotti University, Vallendar, Germany; c Faculty of Human Sciences, Division of Biological Psychology and Neuroscience, Vinzenz Pallotti University, Vallendar, Germany; d Clinical Institute for Psychosomatic Medicine and Psychotherapy, University Hospital Duesseldorf, Duesseldorf, Germany; e Department of Cardiac Surgery, Medical Faculty and University Hospital Duesseldorf, Heinrich-Heine-University, Duesseldorf, Germany; f Cardiovascular Research Institute Duesseldorf (CARID), Medical Faculty, Heinrich-Heine-University, Duesseldorf, Germany; g Department of Psychosomatic Medicine and Psychotherapy, LVR-Hospital Duesseldorf, Hospital of the Heinrich Heine University Düsseldorf, Duesseldorf, Germany

**Keywords:** Cardiac surgery, Depressive symptoms, PHQ-2, Inflammation, CRP, Length of stay

## Abstract

**Introduction:**

Psychological distress is common among patients undergoing cardiac surgery and may influence postoperative recovery and inflammation. This retrospective study examined whether preoperative depressive symptoms predict the postoperative inflammatory response and the length of hospital stay (LOS) in elective cardiac surgery patients.

**Methods:**

Routine clinical data from 951 individuals treated at the Department of Cardiac Surgery, University Hospital Düsseldorf between August 2021 and December 2023 were analyzed. All patients completed the PHQ-4 screening questionnaire prior to surgery, yielding PHQ-2 (depressive symptoms) and GAD-2 (anxiety) subscores. Early and late postoperative C-reactive protein (CRP) indices were derived from routinely collected laboratory measurements. Multivariable regression models adjusted for age, sex, left ventricular ejection fraction, type of surgery, baseline CRP, and anxiety symptoms were estimated, followed by a parallel mediation model to test whether postoperative CRP responses mediated the association between depressive symptoms and postoperative LOS.

**Results:**

Higher preoperative depressive symptoms significantly predicted longer postoperative LOS. They also independently predicted both early and late CRP responses, indicating heightened postoperative inflammation. In the mediation model, late postoperative CRP—but not early CRP—showed a positive association with LOS. Depressive symptoms remained directly associated with prolonged LOS even after accounting for inflammatory pathways, suggesting both direct and indirect mechanisms linking psychological distress to recovery trajectories.

**Conclusion:**

These findings show that preoperative depressive symptoms are linked to postoperative inflammation and recovery. Even brief 2-item measures provides clinically relevant prognostic information. Incorporating such concise psychological screenings into perioperative care could enhance risk stratification and support targeted interventions.

## Highlights

• Depressive symptoms predicted longer hospital stay after cardiac surgery.
• Depressive symptoms were associated with higher postoperative CRP levels.
• Late—but not early—postoperative inflammation was linked to delayed recovery.
• Inflammation partly mediated the link between depression and recovery.
• Brief depression screening showed prognostic value in surgical patients.

## 1 Introduction

Cardiovascular diseases are a major cause of morbidity and mortality, and many affected individuals undergo cardiac surgery (Wang et al., 2016). Such procedures pose both physiological and psychosocial challenges, with depressive symptoms being both preoperatively and postoperatively and associated with poorer postoperative outcomes, underscoring the value of systematic preoperative screening (Blumenthal et al., 2003; Shen et al., 2022; Penninx et al., 2001; Lespérance et al., 2002; Suzuki et al., 2016).

Accumulating evidence indicates that such psychological factors may meaningfully shape postoperative recovery (Salzmann et al., 2020), and mental comorbidity in cardiac patients has been linked to longer hospital stays and more complicated courses (Carter et al., 2016). Psychological interventions delivered prior to surgery can yield clinically relevant benefits, including improved quality of life, reduced postoperative limitations, and attenuated inflammatory responses (Rief et al., 2017). Baseline inflammatory status may also shape responsiveness to such interventions (Salzmann et al., 2021). Emerging evidence further suggests that psychological distress before surgery is associated with altered inflammatory responses afterward. Iin particular, preoperative depressive symptoms have been linked to elevated postoperative C-reactive protein (CRP) levels, suggesting a potential psychobiological pathway influencing recovery (Von Känel et al., 2022; Poole et al., 2014). Serum CRP concentrations have also been found to be elevated in a substantial proportion of patients with major depressive disorder (Wium-Andersen et al., 2013; Osimo et al., 2019, 2020; Pitharouli et al., 2021).

Despite these insights, the mechanisms through which depressive symptoms exert their influence remain insufficiently understood. Postoperative inflammation has been proposed as a key mediator. Prior research emphasizes the temporal dynamics of CRP activation, with persistent rather than immediate postoperative elevations emerging as the most meaningful predictor of impaired recovery: Persistent CRP has been shown to mediate the association between preoperative depressive symptoms and postoperative length of stay following coronary artery bypass grafting (Poole et al., 2014), whereas other studies have identified persistent CRP as a robust predictor of length of stay even in the absence of a direct depressive-symptom effect on CRP or length of stay (Ivankovic et al., 2022). Inconsistent findings in smaller samples underscore the need for adequately powered studies (Buschmann et al., 2025).

Most prior studies used longer depression measures and focused on bypass cohorts (Poole et al., 2014; Ivankovic et al., 2022), leaving the utility of brief tools like the PHQ-2 in broader cardiac surgery samples insufficiently examined. The PHQ-2 is widely used in routine care, yet its prognostic value in this setting remains unclear (Kroenke et al., 2009).

The present study investigates whether preoperative depressive symptoms assessed with the PHQ-2 predict postoperative inflammatory responses and postoperative length of stay in a large cohort of elective cardiac surgery patients. By distinguishing early and late postoperative CRP trajectories and applying robust multivariable and mediation analyses, this study aims to further elucidate the psychobiological mechanisms linking depressive symptoms with postoperative recovery.

## 2 Methods

This study is a clinical retrospective study. Paper-based questionnaires (PHQ-4) collected as part of routine care from elective cardiac surgery patients between 1st of August 2021 and 31st of December 2023 were pseudonymized and transferred into an electronic database. Once all available data had been collected, the dataset was anonymized prior to analysis. The study protocol was reviewed and approved by the Institutional Review Board of Heinrich Heine University Düsseldorf and University Hospital Düsseldorf, Germany, which waived the requirement for informed consent (No. 2024-2740).

The Patient Health Questionnaire-4 (PHQ-4) is a validated instrument for assessing anxiety and depressive symptoms [12]. It consists of four items rated on a 4-point Likert scale (0 = not at all to 3 = nearly every day). The first two items form the PHQ-2 subscale assessing core depressive symptoms, while the last two items constitute the GAD-2 subscale assessing anxiety. Subscale scores range from 0 to 6 each. A PHQ-2 score ≥3 indicates clinically relevant depressive symptoms, and a GAD-2 score ≥3 indicates clinically relevant anxiety. Although the PHQ-4 total score (0–12) can be categorized into normal (0–2), mild (Shen et al., 2022; Penninx et al., 2001; Lespérance et al., 2002), moderate (Suzuki et al., 2016; Salzmann et al., 2020; Carter et al., 2016), and severe (Rief et al., 2017; Salzmann et al., 2021; Von Känel et al., 2022; Poole et al., 2014) symptom levels, the present study focused specifically on the PHQ-2 as the primary measure of preoperative depressive symptoms. The PHQ-4 was administered as part of routine preoperative inpatient assessment. The median interval between hospital admission and surgery was 1 day (mean = 2.07, SD = 2.52; range 0–39 days), indicating that most patients completed the questionnaire shortly before surgery, although a small number of patients had substantially longer preoperative hospital stays.

CRP concentrations were measured in the hospital's central laboratory using the CRP4 (4th generation) particle-enhanced immunoturbidimetric assay (Roche Diagnostics, Mannheim, Germany) on a cobas 8000 c701 analyzer. This conventional assay (i.e., not high-sensitivity CRP) has an analytical measuring range of 0.6–350 mg/L. In routine clinical reporting, CRP values were expressed in mg/dL, and values below 0.1 mg/dL were reported as "<0.1 mg/dL". Blood samples were collected one day before surgery and every 24 h after surgery until discharge. We also assessed age, sex, the pump function of the heart (left ventricular ejection fraction (LVEF)) and medical comorbidities.

### 2.1 Statistical analyses

Analyses were conducted in RStudio (Posit, version 2025.09.1) using R and the *lavaan* package. CRP values were log-transformed (log[x+1]) to reduce skewness. Continuous variables—including log-transformed CRP values, postoperative length of stay, LVEF, and questionnaire scores (PHQ-2, GAD-2) were z-standardized. Extreme values were defined as observations exceeding ±3.29 standard deviations from the sample mean (corresponding to approximately p < .001 in a normal distribution). For CRP, this criterion was applied to each individual postoperative measurement before aggregation into early and late response indices. Values exceeding the threshold were treated as missing. The outlier criterion was applied to reduce the influence of extreme observations and was based on statistical rather than biological considerations. Overall, fewer than 0.6% of observations were affected. Specifically, his procedure affected only a small number of cases for postoperative length of stay (n = 14), LVEF (n = 7), and individual CRP measurements (0–19 per time point), while no extreme values were detected for PHQ-2 or GAD-2. CRP values were aggregated into early (CRP1–CRP3) and late (CRP4–CRP10) CRP response indices using the mean of all available values within each window as suggested by previous publications (Poole et al., 2014). Thus, patients contributed to the respective index even when only a subset of measurements within the corresponding time window was available. As a sensitivity analysis, all models were additionally re-estimated without excluding extreme values.

The primary predictor of interest was preoperative depressive symptoms measured with the PHQ-2. Covariates were selected *a priori* and included age, sex, LVEF, type of surgery, baseline CRP, and GAD-2. Outcomes included early postoperative CRP response, late postoperative CRP response, and postoperative length of stay. Missing data were handled using full-information maximum likelihood (FIML) under a missing-at-random assumption. Statistical significance was assumed if p < .05. Three multivariable regression models were estimated to examine whether PHQ-2 scores predicted (i) postoperative length of stay, (ii) early CRP response, and (iii) late CRP response. Late CRP was modeled without adjustment for early CRP to estimate the total association between PHQ-2 and late postoperative inflammation, given that early CRP represents an intermediate step in the inflammatory trajectory. In a sensitivity analysis, we repeated this model with additional adjustment for early CRP. Standardized and unstandardized coefficients, and 95% confidence intervals were reported. A parallel mediation model was then estimated to evaluate whether early and late postoperative CRP responses mediated the association between PHQ-2 and postoperative length of stay, adjusting for all covariates. Across participants, an average of 2.71 early CRP measurements contributed to the early CRP index (median = 3; range: 0–3), indicating that most individuals provided complete early postoperative CRP data. For the late CRP index, an average of 4.05 measurements contributed to the aggregated value (median = 4; range: 0–7), reflecting the larger number of potential postoperative assessment points and greater variability in measurement availability. This approach allowed all available information to be used while accommodating routine missingness in clinical postoperative CRP trajectories.

## 3 Results

Patients' baseline characteristics are displayed in Table 1. Patients had a mean age of 65.3 years (SD = 10.7), and the majority were male (75.1%). Mean left ventricular ejection fraction was 0.53 (SD = 0.07). Preoperative depressive and anxiety symptoms were generally low (PHQ-2: M = 1.26, SD = 1.62; GAD-2: M = 1.54, SD = 1.63). Higher preoperative depressive symptoms were weakly associated with higher baseline CRP concentrations (Pearson's r = 0.133, 95% CI [0.069, 0.196], p < .001). A similar but smaller association was observed for anxiety symptoms (r = 0.075, 95% CI [0.011, 0.139], p = .022).

**Table 1:** Baseline characteristics (N = 951).

Variable	
Age (years), mean ± SD	65.32 ± 10.69
Sex, n (%)	
Male	714 (75.1%)
Female	237 (24.9%)
Left ventricular ejection fraction (LVEF), mean ± SD	0.53 ± 0.07
Depressive symptoms (PHQ-2), mean ± SD	1.26 ± 1.62
Anxiety symptoms (GAD-2), mean ± SD	1.54 ± 1.63
Surgery type, n (%)	
Aortic replacement	55 (5.8%)
Coronary bypass	410 (43.1%)
Valve repair	158 (16.6%)
Valve replacement	306 (32.2%)
Other	22 (2.3%)
Hypertension, n (%)	
No	343 (36.1%)
Yes	608 (63.9%)
Coronary artery disease, n (%)	
No	330 (34.7%)
Yes	621 (65.3%)
Dyslipidemia, n (%)	
No	589 (61.9%)
Yes	362 (38.1%)
History of mental disorder, n (%)	
No	899 (94.5%)
Yes	52 (5.5%)
Diabetes mellitus, n (%)	
No	745 (78.3%)
Yes	206 (21.7%)
Antidepressant medication, n (%)	
No	879 (94.5%)
Yes	51 (5.5%)
Statin therapy, n (%)	
No	225 (24.2%)
Yes	705 (75.8%)
Baseline CRP in mg/dL, mean ± SD	0.50 ± 1.39
Postoperative length of stay (days), mean ± SD	5.14 ± 5.56

Note. Continuous variables are mean ± SD; categorical variables are n (%).

Regarding surgical procedures, 43.1% underwent coronary bypass surgery and 32.2% valve replacement, while 16.6% received valve repair and 5.8% aortic replacement. Cardiovascular comorbidity was frequent: 63.9% had hypertension, 65.3% coronary artery disease, 38.1% dyslipidemia, and 21.7% diabetes mellitus. A documented history of mental disorder and antidepressant use were each present in 5.5% of patients. Statin therapy was common (75.8%). Mean preoperative CRP concentration was 0.50 mg/dL(SD = 1.39). The mean postoperative length of stay on the normal ward was 5.14 days (SD = 5.56). The late CRP index was available for 930 of 951 patients (97.8%). Only 21 patients (2.2%) had no CRP measurements during the late postoperative period (days 4–10) and therefore could not contribute to analyses involving late CRP.

### 3.1 PHQ-2 predicts length of stay, early and late CRP response

Higher preoperative depressive symptoms (PHQ-2) were significantly associated with a longer postoperative hospital stay (B = 0.300, 95% CI [0.103, 0.497], p = .003; Table 2), independent of age, sex, LVEF, type of surgery, baseline CRP, and anxiety symptoms.

**Table 2:** Associations of preoperative depressive symptoms (PHQ-2) with postoperative outcomes adjusted for age, sex, pump function (LVEF), type of surgery, preoperative CRP, and GAD-2.

Outcome	Predictor	B	95% CI	β	p
Length of stay	**Age**	**0.048**	**[0.025, 0.071]**	**0.147**	**< 0.001**
	**Sex**	**0.635**	**[0.074, 1.197]**	**0.079**	**0.027**
	LVEF	−0.034	[-0.073, 0.005]	−0.062	0.089
	**Surgery: CABG vs. Aortic Replacement**	**1.099**	**[0.043, 2.155]**	**0.074**	**0.041**
	**Surgery: CABG vs. Valve Replacement**	**0.900**	**[0.412, 1.389]**	**0.121**	**< 0.001**
	**Surgery: CABG vs. Valve Reconstruction**	**1.684**	**[0.986, 2.381]**	**0.181**	**< 0.001**
	**Surgery: CABG vs. Other**	**−1.605**	**[-2.738, -0.472]**	**−0.070**	**0.006**
	**Preoperative CRP**	**1.740**	**[0.592, 2.887]**	**0.133**	**0.003**
	GAD-2	−0.073	[-0.268, 0.123]	−0.034	0.465
	**PHQ-2**	**0.300**	**[0.103, 0.497]**	**0.140**	**0.003**
Early CRP response	Age	**−0.001**	**[-0.003, 0.001]**	**−0.027**	**0.400**
	Sex	−0.008	[-0.068, 0.052]	−0.008	0.805
	LVEF	0.001	[-0.003, 0.006]	0.018	0.617
	Surgery: CABG vs. Aortic Replacement	0.012	[-0.105, 0.128]	0.007	0.845
	Surgery: CABG vs. Valve Replacement	0.038	[-0.025, 0.100]	0.044	0.235
	Surgery: CABG vs. Valve Reconstruction	−0.004	[-0.069, 0.061]	−0.004	0.905
	**Surgery: CABG vs. Other**	**−0.450**	**[-0.696, -0.203]**	**−0.169**	**< 0.001**
	Preoperative CRP	0.324	[0.214, 0.434]	0.214	<0.001
	GAD-2	−0.009	[-0.032, 0.015]	−0.036	0.461
	**PHQ-2**	**0.029**	**[0.006, 0.051]**	**0.115**	**0.012**
Late CRP response	**Age**	**0.005**	**[0.002, 0.009]**	**0.110**	**< 0.001**
	Sex	−0.037	[-0.117, 0.044]	−0.030	0.370
	LVEF	−0.004	[-0.009, 0.001]	−0.050	0.114
	**Surgery: CABG vs. Aortic Replacement**	**0.415**	**[0.284, 0.546]**	**0.182**	**< 0.001**
	**Surgery: CABG vs. Valve Replacement**	**0.184**	**[0.106, 0.262]**	**0.161**	**< 0.001**
	Surgery: CABG vs. Valve Reconstruction	−0.028	[-0.123, 0.067]	−0.020	0.564
	Surgery: CABG vs. Other	−0.172	[-0.366, 0.021]	−0.049	0.080
	**Preoperative CRP**	**0.480**	**[0.341, 0.619]**	**0.239**	**< 0.001**
	**GAD-2**	**−0.039**	**[-0.068, -0.009]**	**−0.118**	**0.010**
	**PHQ-2**	**0.048**	**[0.020, 0.075]**	**0.144**	**< 0.001**

*Note.* Predictors with *p* < .05 are shown in bold. Sex was coded as female = 0 and male = 1. The five surgery types were dummy-coded with CABG serving as the reference category.

PHQ-2 scores also significantly predicted both components of the postoperative inflammatory response. Higher depressive symptoms were associated with a greater early CRP response (B = 0.029, 95% CI [0.006, 0.051], p = .012) as well as a greater late CRP response (B = 0.048, 95% CI [0.020, 0.075], p < .001), after adjustment for all covariates. Preoperative CRP concentrations were also strongly associated with postoperative inflammatory responses. Higher baseline CRP predicted greater early postoperative CRP levels (B = 0.324, 95% CI [0.214, 0.434], p < .001) and greater late postoperative CRP levels (B = 0.480, 95% CI [0.341, 0.619], p < .001). In a supplementary analysis additionally adjusting for early postoperative CRP, PHQ-2 remained significantly associated with late CRP (B = 0.036, 95% CI [0.011, 0.061], p = .005), indicating that the association between depressive symptoms and sustained postoperative inflammation was not fully explained by the initial inflammatory response.

### 3.2 Mediation analysis

**Fig. 1:**
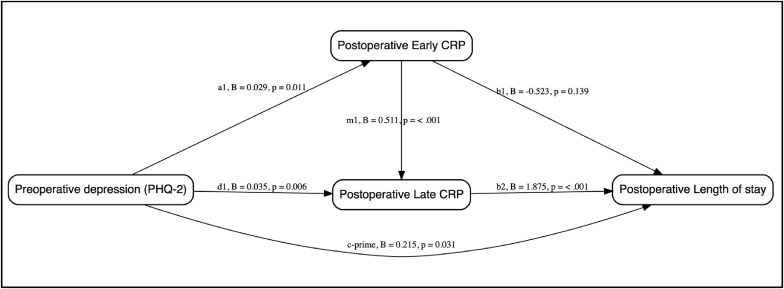
Mediation of the association between preoperative depressive symptoms and postoperative length of stay.

As shown in Fig. 1, preoperative depressive symptoms (PHQ-2) significantly predicted both early postoperative CRP (a_1_ = 0.029, p = .011) and late postoperative CRP (d_1_ = 0.035, p = .006). Early CRP strongly predicted late CRP (m_1_ = 0.511, p < .001). Early CRP was not associated with postoperative length of stay (b_1_ = −0.523, p = .139), whereas late CRP showed a robust positive association with length of stay (b_2_ = 1.875, p < .001). In addition, PHQ-2 remained directly associated with postoperative length of stay (c′ = 0.215, p = .031).

Analysis of indirect effects revealed that the pathway through early CRP alone was not significant (B = −0.015, 95% CI [–0.038, 0.008], p = .190). In contrast, the indirect effect through late CRP was statistically significant (B = 0.066, 95% CI [0.016, 0.116], p = .009). The serial pathway of PHQ-2 → early CRP → late CRP → length of stay—was also significant (B = 0.028, 95% CI [0.004, 0.051], p = .021), indicating that early CRP contributes indirectly by influencing late CRP. The total indirect effect of PHQ-2 on length of stay was significant (B = 0.079, 95% CI [0.025, 0.133], p = .004).

Overall, these findings suggest that sustained postoperative inflammation represents one important pathway linking preoperative depressive symptoms to postoperative recovery.

**Fig. 2:**
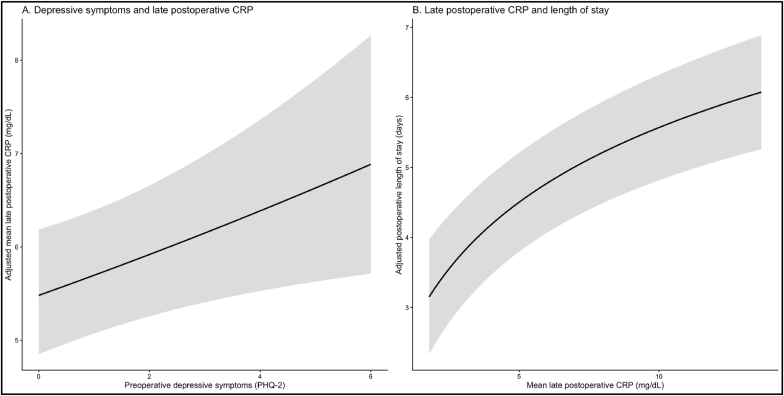
Adjusted associations between depressive symptoms, late postoperative inflammation, and postoperative recovery. Panel A shows average adjusted predictions of mean late postoperative CRP concentrations across the full range of PHQ-2 scores. Panel B shows the association between mean late postoperative CRP concentrations and adjusted postoperative length of stay. Shaded areas represent 95% confidence intervals. Predictions are adjusted for age, sex, left ventricular ejection fraction, anxiety symptoms, baseline CRP, early postoperative CRP, and surgery type.

Fig. 2 illustrates the adjusted associations underlying the mediation model. Higher preoperative depressive symptom severity was associated with progressively higher late postoperative CRP concentrations (Fig. 2A). In turn, elevated late postoperative CRP levels were associated with longer postoperative length of stay (Fig. 2B), supporting the role of sustained postoperative inflammation as a pathway linking psychological distress and recovery.

To facilitate clinical interpretation, average adjusted estimates were additionally derived using the conventional PHQ-2 screening threshold. Patients with PHQ-2 scores <3 had an adjusted mean late postoperative CRP concentration of 5.61 mg/dL (95% CI, 4.99–6.30), whereas those with PHQ-2 scores ≥3 had an adjusted mean concentration of 6.40 mg/dL (95% CI, 5.54–7.39). These values corresponded to adjusted postoperative lengths of stay of 4.67 days (95% CI, 3.97–5.38) and 4.87 days (95% CI, 4.16–5.58), respectively.

Sensitivity analyses performed without excluding extreme values yielded a largely comparable pattern of findings. Associations between depressive symptoms and both early and late postoperative CRP responses remained statistically significant, and the indirect effects via late CRP were preserved. In contrast, the direct association between depressive symptoms and postoperative length of stay was attenuated and no longer reached statistical significance.

## 4 Discussion

The present study indicates that preoperative depressive symptoms assessed with the PHQ-2 are associated with heightened postoperative inflammation and longer hospital stay in a large elective cardiac surgery cohort. Importantly, only late postoperative CRP levels mediated the link between depressive symptoms and prolonged recovery, supporting inflammation as a delayed pathway connecting psychological distress and surgical outcome. This distinction aligns with evidence that CRP reflects a secondary phase of systemic activity; Engler et al. demonstrated that CRP rises well after the IL-6 peak and remains elevated for days, indicating sustained inflammatory tone rather than acute response (Engler et al., 2023).

Our findings mirror studies showing that sustained postoperative inflammation—rather than the immediate post-surgical inflammatory surge—carries prognostic relevance: Poole et al. reported that persistent CRP elevations mediated the association between preoperative depressive symptoms and delayed recovery after CABG [12], while Ivanković et al. similarly identified persistent CRP as a predictor of prolonged hospitalization (Ivankovic et al., 2022). Although these earlier studies relied on the Beck Depression Inventory and smaller CABG-only samples, the current study confirms a comparable pattern using a brief, validated screening tool (PHQ-2) across a broader cardiac surgery population.

Notably, our results contrast with those of Buschmann et al. (2025), who did not observe an association between PHQ-4 and postoperative outcomes. Differences in sample size and analytical depth may account for this discrepancy. In supplementary analyses, a model including the PHQ-4 total score performed similarly to a model including PHQ-2 and GAD-2 separately, with both explaining an identical proportion of variance in postoperative length of stay (R^2^ = 0.09). The PHQ-4 total score was significantly associated with length of stay (β = 0.098, p = .004), whereas in the model including PHQ-2 and GAD-2 separately, depressive symptoms showed a somewhat stronger association (β = 0.140, p = .003) and anxiety symptoms were not independently associated with recovery. These findings suggest that the prognostic information contained in the PHQ-4 may primarily reflect its depressive symptom component. Moreover, the large sample of the present study provided substantially greater statistical power and enabled refined modeling of early and late postoperative inflammatory trajectories, allowing a clearer temporal characterization of the pathways linking depressive symptoms with recovery. Although depressive symptoms showed a direct association with postoperative length of stay in the primary analyses, sensitivity analyses without outlier exclusion suggested that this association was less robust. In contrast, the associations with early and late postoperative CRP responses, as well as the indirect effects via late CRP, remained largely unchanged. These findings support the notion that sustained postoperative inflammation may represent a particularly robust pathway linking psychological distress with recovery. Nevertheless, additional behavioral and physiological mechanisms, such as reduced mobility, diminished treatment engagement, sleep disturbance, hyperglycemia, or autonomic dysregulation, may also contribute and warrant further investigation.Strengths of this study include its large real-world sample, routine clinical assessment of depressive symptoms with an ultra-brief instrument, and use of aggregated CRP indices that utilize all available postoperative measurements. Limitations include the retrospective design, lack of structured mental diagnoses, and the inability to disentangle postoperative inflammatory responses attributable to surgical trauma from those potentially related to psychological factors. An additional limitation is that CRP was assessed using a conventional clinical assay rather than a high-sensitivity assay, potentially limiting the detection of very low-grade inflammatory activity. Values below 0.1 mg/dL were reported as "<0.1"; however, this did not result in missing data and is unlikely to have substantially affected the analyses given the pronounced postoperative CRP elevations observed after cardiac surgery. Additionally, the reliance on PHQ-2 captures core depressive symptoms but not the broader spectrum of psychological distress. Outlier handling was based on statistical rather than biological considerations; however, sensitivity analyses without excluding extreme values yielded comparable findings for the inflammatory pathways, whereas the association with postoperative length of stay appeared less robust.

## 5 Conclusion

This study shows that brief preoperative screening for depressive symptoms has prognostic value in elective cardiac surgery. Higher symptom levels predicted heightened late—but not early—postoperative CRP responses and longer hospital stay, indicating that sustained inflammation may represent a relevant biological pathway linking psychological distress to recovery. Depressive symptoms measured with the PHQ-2 provided information beyond medical risk factors and helped identify patients at elevated risk for prolonged recovery. Incorporating PHQ-2 screening into routine preoperative assessment may therefore improve risk stratification and inform targeted perioperative care.

## Funding sources

None.

## CRediT authorship contribution statement

**Stefan Salzmann:** Conceptualization, Data curation, Formal analysis, Methodology, Writing – original draft, Writing – review & editing. **Frank Euteneuer:** Formal analysis, Writing – review & editing. **Ruben Domroese:** Investigation, Writing – review & editing. **Udo Boeken:** Resources, Writing – review & editing. **Artur Lichtenberg:** Resources, Writing – review & editing. **Ulrike Dinger:** Resources, Writing – review & editing. **Clarissa Zimmermann:** Investigation, Writing – review & editing. **Malin Neumann:** Investigation, Writing – review & editing. **Ralf Schäfer:** Resources, Writing – review & editing. **Anna Markser:** Conceptualization, Formal analysis, Writing – original draft, Writing – review & editing.

## Declaration of competing interest

The authors declare that they have no known competing financial interests or personal relationships that could have appeared to influence the work reported in this paper.

## Data Availability

Data will be made available on request.
